# The need for pharmacotherapy among patients with one or more elevations on glucose tolerance test

**DOI:** 10.1210/clinem/dgag062

**Published:** 2026-02-12

**Authors:** Lizelle Comfort, Alexis Benjamin, Emma Brenner, David Krantz, Clarissa Bonanno, Heather Levin, Alefiya Faizullabhoy, Burton Rochelson

**Affiliations:** Department of Obstetrics and Gynecology, Division of Maternal Fetal Medicine, Northwell, New Hyde Park, NY 11042, USA; Zucker School of Medicine at Hofstra/Northwell, Hempstead, NY 11549, USA; Department of Obstetrics and Gynecology, Division of Maternal Fetal Medicine, Northwell, New Hyde Park, NY 11042, USA; Department of Obstetrics and Gynecology, Division of Maternal Fetal Medicine, Northwell, New Hyde Park, NY 11042, USA; Department of Obstetrics and Gynecology, Division of Maternal Fetal Medicine, Northwell, New Hyde Park, NY 11042, USA; Department of Obstetrics and Gynecology, Division of Maternal Fetal Medicine, Northwell, New Hyde Park, NY 11042, USA; Department of Obstetrics and Gynecology, Division of Maternal Fetal Medicine, Northwell, New Hyde Park, NY 11042, USA; Department of Obstetrics and Gynecology, Division of Maternal Fetal Medicine, Northwell, New Hyde Park, NY 11042, USA

**Keywords:** glucose intolerance, gestational diabetes, glucose testing in pregnancy, pharmacologic treatment, prediction, risk stratification, treatment modality

## Abstract

**Objective:**

To identify factors associated with need for pharmacotherapy among patients with glucose intolerance and 1 elevation on 3-hour oral glucose tolerance test (OGTT).

**Methods:**

Retrospective cohort study of singleton gestations 24 weeks or greater with at least 1 elevation on OGTT. The primary outcome was need for pharmacotherapy for glycemic control. To evaluate timing of OGTT elevation with need for pharmacotherapy, a logistic regression model controlled for maternal race/ethnicity, body mass index (BMI), gestational age at time of OGTT, age, parity, history of gestational diabetes, and history of large neonate. The need for pharmacotherapy was assessed based on number and extent of testing elevations. A predictive model based on linear discriminant analysis was developed.

**Results:**

Overall, 480 patients had 1 OGTT elevation; of these, 19.2% required pharmacotherapy. Fasting elevations were most associated with development of medication-requiring diabetes. A predictive model for risk of pharmacotherapy in patients with an abnormal OGTT based on BMI and extent of elevation at each OGTT time point increased the identification of patients requiring pharmacotherapy by 15.4%. At least 2 OGTT elevations occurred in 376 patients; these patients were more likely to require pharmacotherapy for glycemic control compared to those with 1 elevation. Patients with increasing BMI values had increased need for pharmacotherapy regardless of the number of abnormal values.

**Conclusion:**

Among patients with 1 OGTT elevation, fasting elevation and BMI are predictive of need for anti-glycemic medications. Predictive models may be useful in assessing need for pharmacotherapy for patients with abnormal OGTT, not otherwise meeting criteria for gestational diabetes.

Glucose intolerance (defined as an abnormal glucose challenge test [GCT] with 1 of 4 elevations on 3-hour oral glucose tolerance test [OGTT]) is associated with known obstetrical complications (1, 2). A large meta-analysis demonstrated adverse outcomes (specifically, increased risk of macrosomia/large for gestational age, neonatal hypoglycemia, cesarean delivery, pregnancy-induced hypertension) based on glucose; however, this study excluded patients who were treated based on 1 abnormal value (3). In this meta-analysis, risks of glucose intolerance were similar to risks of patients with gestational diabetes. Despite observed risks and suggested benefits from treatment, current guidelines do not support universal treatment of patients with an abnormal OGTT who do not meet nationally accepted criteria for gestational diabetes.

Predictive GCT values have previously been studied. A threshold of 200 mg/dL or more on 1-hour GCT confers a positive predictive value of 50% to 80% for development of gestational diabetes (4, 5). However, no threshold GCT value under 199 alone reliably predicts gestational diabetes, although increasing values are associated with increased positive predictive value (6). One retrospective analysis examined outcomes among patients who ruled in for gestational diabetes based on one of the following: 1-hour GCT value of at least 200, at least 2 of 4 elevated values on OGTT, or glucose intolerance (based on 1 of 4 elevated values on OGTT); this study found that macrosomia and large for gestational age incidence were highest in the group with 1 elevated OGTT value; in addition, hospitalization rates, hypoglycemia, hyperbilirubinemia, and polycythemia were more common in neonates born to mothers with 1 elevated OGTT value and to mothers with a GCT of 200 mg/dL (4). This study demonstrated that there is a critical threshold of testing that may be associated with poorer perinatal outcomes. On the other hand, one prospective study that stratified outcomes based on the number of elevations on OGTT found that an increasing number of abnormal glucose values on OGTT is associated with increased risk of adverse outcomes. All groups with elevations on OGTT had elevated risk of large-for-gestational-age neonates (7).

Current OGTT thresholds are extrapolated from those established by O'Sullivan and Mahan (8). When current thresholds (those proposed by Carpenter-Coustan and the National Diabetes Data Group) were compared in a randomized control trial, treatment benefit did not vary by the diagnostic criteria met (9). Multiple retrospective abstracts have examined whether the timing of OGTT elevations is associated with clinical outcomes; one study demonstrated that elevations in the 1-hour postprandial values on OGTT were associated with a higher mean birth weight, neonatal hypoglycemia, and labor disorders, whereas elevations in the fasting value on OGTT were associated with increased rates of neonatal intensive care unit (NICU) admission (10). Conversely, another study did not demonstrate difference in subsequent glycemic control among different time elevations (11).

The primary objective of this study is to retrospectively compare the need for pharmacotherapy among patients with glucose intolerance (defined as having 1 elevation on OGTT) at 24 to 28 weeks' gestation compared to patients with gestational diabetes (at least 2 OGTT elevations). The secondary objective is to identify predictive factors associated with development of medication-requiring gestational diabetes among these groups, including the degree of normality (the extent and number of elevations on OGTT).

## Methods

This study included patients with singleton gestations and at least 1 elevation on 3-hour OGTT performed at 24 to 28 weeks of gestation or later. Only patients with full OGTT data were included. Patients with pregestational diabetes (those having hemoglobin A1C exceeding 6.4% or having a known diagnosis of diabetes prior to pregnancy), early diagnosis of gestational diabetes, or glucose intolerance (defined as abnormality on OGTT prior to 24 weeks' gestation), normal glucose screen or no elevations on OGTT, significant fetal anomaly or fetal demise, and multifetal gestations were excluded. The outpatient electronic medical record system ASOBGYN was used to search for patients who met inclusion criteria using the following search items “*glucose intolerance*,” “*abnormal glucose*,” and “*gestational diabetes*” between January 2015 and July 2023. The study received institutional review board approval prior to data collection.

At the study institution, 1-hour GCT is routinely performed with a 50-gram glucose solution. When GCT value is greater than or equal to 135 mg/dL, a 3-hour OGTT is performed with a 100-gram glucose solution, and glucose values are collected prior to administration when patients are fasting and at 1-hour increments following OGTT consumption. Carpenter-Coustan criteria were used for OGTT thresholds (9). If glucose screen is greater than or equal to 180 mg/dL, a 3-hour OGTT is deemed unnecessary, and the patient is considered to have gestational diabetes. The study institution treats all patients with 1 or more elevations on OGTT as having gestational diabetes given the literature suggesting similar outcomes to patients with 2 or more elevations on OGTT (3) and studies that demonstrate improved identification of patients at risk for adverse pregnancy outcome with more conservative diagnostic criteria (12). Patients are scheduled with a certified diabetes educator as soon as an abnormal GTT is obtained and are given guidelines regarding blood glucose goals and taught blood glucose monitoring. Follow-up visits will be scheduled approximately every 1 to 4 weeks depending on glycemic control. At each visit, the patient's diet, home glucose monitoring log, and management problems are reviewed by the diabetes educator and discussed with a physician.

Meal planning is emphasized as the key to glycemic control. This diet is based on caloric requirements for singleton gestations of 30 to 35 calories/kg/day as determined by desired body weight (13). There is a consistent amount of carbohydrate calories distributed throughout the meal and snack pattern with a minimum of 3 meals and an afternoon and bedtime snack. A morning snack may also be added for patients on insulin therapy. Patients are given a copy of an individually formulated meal plan.

Patients are instructed or reassessed in capillary blood glucose monitoring skills using a glucometer and testing. Following receipt of a personal glucometer, calibration is performed; the Food and Drug Administration guidelines for personal use glucose meters require: 95% within +/− 15% across the measuring range, and 99% within +/− 20% across the measuring range (14). Women with problems on the degree of accuracy of their glucometer are advised to use a different meter. In 2024, the institutional protocol was updated to incorporate the use of continuous glucose monitors as an adjunct to blood glucose monitoring; however, patients in the program are advised to continue to use personal glucometer for fasting and 1-hour postprandial values.

Insulin therapy is typically initiated when home blood glucose monitoring reveals a pattern of fasting glucose level greater than 95 mg/dL and/or 1-hour postprandial glucose levels greater than 140 mg/dL. Options for medical therapy include insulin therapy or oral anti-hyperglycemic agents, with insulin being the first line of medication treatment. Generally, initial insulin therapy is administered as a regimen of intermediate, long-acting, and/or rapid acting insulin. In the institutional protocol, there is no absolute percentage of poorly controlled glucose values that warrant initiation of medical therapy; however, patients are typically initiated on therapy once reviewed values are elevated. For example, if there is a pattern suggesting 30% time-based elevations above the aforementioned thresholds that cannot be attributed to dietary choices alone, pharmacotherapy is initiated.

Variables including maternal age, race/ethnicity, pre-pregnancy body mass index (BMI), gestational weight gain, parity, history of gestational diabetes in prior pregnancy, history of macrosomia, 1-hour GCT value, 3-hour OGTT values, and gestational age at time of glucose screening were retrospectively collected via individual review of the institutional electronic medical record system. Outcomes including need for pharmacotherapy (with metformin, glyburide, and/or insulin), gestational age at delivery rounded to the completed gestational week, delivery mode, hypertensive disorder of pregnancy, birth weight, and composite neonatal complications including NICU admission, stillbirth and/or intrauterine fetal demise, shoulder dystocia, neonatal hypoglycemia, and hyperbilirubinemia were recorded.

The primary outcome was the need for pharmacotherapy. Our aims were: 1) to identify factors associated with need for pharmacotherapy among patients with only one OGTT elevation; 2) to compare the need for pharmacotherapy among patients with 1 OGTT elevation compared to patients with 2 or more OGTT elevations; 3) to compare antepartum and neonatal outcomes (including neonatal birth weight, polyhydramnios, NICU admission, cesarean delivery, shoulder dystocia, 5-minute Apgar score) among patients with 1 OGTT elevation compared to patients with 2 or more OGTT elevations; and 4) to develop a prediction model for need for pharmacotherapy among patients with 1 or more OGTT elevation.

Continuous variables were compared using Wilcoxon Rank-Sum test and categorical variables were compared using Chi-Square. We built 3 logistic regression models; each of the models controlled for maternal race/ethnicity, BMI, gestational age at OGTT, age, parity, history of gestational diabetes, and history of a neonate born large for gestational age. The first model evaluated patients with 1 abnormal OGTT result and compared the odds of requiring pharmacotherapy based on which of the 4 OGTT values was abnormal. The second model used all patients and compared patients with only 1 elevation on OGTT to patients with 2 or more elevations on OGTT. The third model evaluated all patients and evaluated odds based on the number of abnormal OGTT values. In addition to odds ratios, relative risks and confidence intervals using nonparametric bootstrapping were calculated to avoid overstating the effect of OGTT (15).

Finally, a model to determine the risk of requiring pharmacotherapy based on the OGTT concentration values at each of the 4 time points and BMI was developed using linear discriminant analysis. All OGTT values were corrected for degree of normality by subtracting the cutoff value from the observed value. Reference parameters for this approach were calculated by using nonparametric statistics. The mean was estimated via the median, the SD estimated by the difference between the 90th and 10th percentile divided by 2.563 and correlations estimated using spearman rank correlation coefficients. Screening performance was evaluated using 5-fold cross-validation to reduce bias from overfitting. Calibration was performed by comparing the observed incidence of pharmacotherapy and its associated CI in each of the 10 deciles of observed risk with the estimated risk from the model. The incidence of pharmacotherapy in this study was used as a priori risk in the analysis and applied uniformly to all subjects. In determining the reference data, results from both the patients with 1 elevation and those with 2 or more elevations on OGTT were utilized. Case-wise deletion was used to exclude any record with missing information on any variable from a particular analysis.

## Results

We included 856 patients: 480 patients with 1 OGTT elevation and 376 patients with at least 2 elevations on OGTT. Patients with 1 OGTT elevation had a lower BMI (26.4 vs 28.9), lower 1-hour GCT value, and were less likely to have a history of gestational diabetes compared to patients with 2 or more OGTT elevations (Table 1). For patients with only 1 elevation on OGTT, 5 patients were underweight (BMI less than 18.5), 166 patients were ideal weight (BMI 18.5-24.9), 143 patients were overweight (BMI 25-29.5), 98 patients had class 1 obesity (BMI 30.0-34.9), 43 patients had class 2 obesity (BMI 35.0-39.9), and 19 patients had class 3 obesity (BMI greater than or equal to 40). For patients with 2 or more elevations on OGTT, 2 patients were underweight (BMI less than 18.5), 95 patients were ideal weight (BMI 18.5-24.9), 116 patients were overweight (BMI 25-29.5), 86 patients had class 1 obesity (BMI 30.0-34.9), 44 patients had class 2 obesity (BMI 35.0-39.9), and 22 patients had class 3 obesity (BMI greater than or equal to 40).

**Table 1:** Demographics for patients with only 1 elevation and 2 or more elevations on OGTT

Characteristic	1 abnormal test [N = 480]	2-plus abnormal tests [N = 375]	P*
Maternal age at delivery, median (IQR)	33 (30, 36) [N = 468]	33 (30, 37) [N = 344]	.417
Pre-pregnancy BMI, median (IQR)	27 (23, 32) [N = 474]	29 (25, 33) [N = 365]	<.001
Race/ethnicity	[N = 440]	[N = 355]	.205
White	45.5%	41.4%	
Black	6.8%	11.3%	.023
Hispanic	20.0%	21.4%	.397
Asian	20.0%	17.8%	.894
Other/multiracial	7.7%	8.2%	.588
Parity, median (IQR)	1 (0, 1) [N = 480]	1 (0, 2) [N = 361]	.027
GCT result (mg/dL), median (IQR)	151 (142, 162) [N = 405]	159 (147, 173) [N = 306]	<.001
Gestational age (completed weeks) at time of OGTT, median (IQR)	28 (26, 29)	28 (27, 30)	.061
Prior gestational diabetes	36/480 (7.5%)	63/359 (17.5%)	<.001
Prior macrosomia (neonatal weight >90th percentile for gestational age)	26/451 (5.8%)	28/343 (8.2%)	.184

Abbreviations: BMI, body mass index, in kg/m^2^; GCT, 1-hour glucose challenge test; IQR, interquartile range; OGTT, 3-hour glucose tolerance test.

Pharmacologic treatment patterns differed by OGTT elevation group. Among patients requiring pharmacotherapy with 1 OGTT elevation, 65 received insulin: 50 (58.0%) basal insulin only, 7 (8.0%) basal-bolus, and 7 (8.0%) bolus-only insulin. Additionally, 16 patients (18.2%) were treated with metformin, 5 (5.7%) with glyburide, and 1 with a combination of insulin and metformin. A total of 96 patients with 2 or more OGTT elevations received insulin, including 70 (44.8%) on basal insulin only, 20 (12.8%) on basal-bolus therapy, and 6 (3.8%) on bolus-only insulin. Metformin was used by 33 patients (21.2%), glyburide by 22 (14.1%), and 2 patients received combined insulin and metformin therapy. The patients who took glyburide were predominantly from earlier in the study cohort timeframe.

Of patients with only 1 elevation on OGTT, 59 had an elevated fasting value, 247 elevated 1-hour, 140 elevated 2-hour, and 34 elevated 3-hour value. Overall, 19.2% of total patients with 1 elevation on OGTT required pharmacotherapy. Those who required pharmacotherapy were more likely to have a higher BMI and to have a history of gestational diabetes in a prior pregnancy (Table 2). The rate at which patients with only 1 OGTT elevation required pharmacotherapy varied based on the timing of the variation (*P* < .001), with rates highest for patients with fasting elevation (39%) compared to the other time points (17.8%, 15%, and 11.8%, for 1-hour, 2-hour, and 3-hour elevation, respectively). Using logistic regression (Table 3), there was still a significant difference between testing time points after controlling for covariates. Converting the odds ratios to relative risks demonstrated adjusted relative risks (95% CI) of 0.48 (0.31, 0.76), 0.40 (0.22, 0.68) and 0.26 (<0.01, 0.60) for 1-hour, 2-hour, and 3-hour timepoints, respectively. Five patients with 1 elevation on OGTT required finger stick glucose monitoring to diagnose 1 patient with medication-requiring diabetes. Among the 376 patients with 2 or more elevations on OGTT, 228 patients had 2 elevated measurements, 110 had 3 elevated measurements, and 38 had all 4 elevated measurements. Among patients with at least 2 elevations on OGTT, 42% required pharmacotherapy. These patients were more likely to require pharmacotherapy for glycemic control compared to those with 1 elevation (adjusted OR 2.89 CI 2.05, 4.08, with associated adjusted RR 2.18 CI 1.71, 2.79). However, patients with only 1 elevation were more likely to develop some obstetric complications compared to patients with 2 or more elevations (Table 4). Specifically, polyhydramnios was significantly more common among patients with only 1 elevation on OGTT compared with those with at least 2 elevations on OGTT in the overall analysis (RR 2.64, 95% CI 1.27-5.47; *P* = .009). This association was not statistically significant when stratified by treatment type, with relative risks were similar between dietary management alone and pharmacotherapy groups (Equal RR *P* = .842). A composite of neonatal complications also occurred more frequently in patients with only 1 OGTT elevation overall (23.5% vs 16.0%; RR 1.47, 95% CI 1.09-1.98); this finding was significant for those managed with diet alone but was not significant for those requiring pharmacotherapy. There was no difference in maternal hypertensive disorders or incidence of large-for-gestational-age neonates when comparing these groups.

**Table 2:** Demographics of patients with only 1 elevation on glucose tolerance test

Characteristic	Medication = No	Medication = Yes	*P* value
Maternal age at delivery, median (IQR)	33 (30, 36) [N = 378]	34 (31, 36) [N = 90]	.204
BMI (pre-pregnancy), median (IQR)	26.4 (23.0, 30.3) [N = 382]	30.8 (25.5, 34.9) [N = 92]	<.001
Race/ethnicity	[N = 355]	[N = 85]	.590
White	45.9%	43.5%	
Black	7.0%	5.9%	.809
Hispanic	20.6%	17.7%	.768
Asian	19.7%	21.2%	.698
Other/multiracial	6.8%	11.8%	.142
Parity, median (IQR)	1 (0, 1) [N = 388]	1 (0, 2) [N = 92]	.927
GCT result, median (IQR)	152 (142, 162) [N = 328]	151 (142, 163) [N = 77]	.935
Gestational age at OGTT, median (IQR)	28 (26, 29) [N = 388]	28 (26, 29) [N = 92]	.270
Prior gestational diabetes	6.2% [N = 388]	13.0% [N = 92]	.025
Prior macrosomia	5.5% [N = 365]	7.0% [N = 86]	.608
Birth weight (grams), median (IQR)	3288 (2940, 3590) [N = 358]	3270 (2945, 3650) [N = 85]	.962
Cesarean delivery	43.6% [N = 358]	48.2% [N = 85]	.437
Gestational age at delivery, median (IQR)	39 (38, 39) [N = 358]	38 (37, 39) [N = 85]	.001
Weight gain in pregnancy, median (IQR)	26 (18, 35) [N = 372]	24 (16, 34) [N = 87]	.122

Abbreviations: BMI, body mass index; GCT, 1-hour glucose challenge test; IQR, interquartile range; OGTT, 3-hour glucose tolerance test.

**Table 3:** Adjusted odds ratios for outcome by OGTT abnormality and clinical covariates

		Model (N = 417)		Model (N = 743)		Model (N = 743)	
Predictor/Metric	Category	aOR (95% CI)	*P*	aOR (95% CI)	*P*	aOR (95% CI)	*P*
Intercept		0.40 (95% CI: 0.20, 0.81)	.011	0.19 (95% CI: 0.13, 0.27)	<.001	0.19 (95% CI: 0.14, 0.27)	<.001
OGTT	OGTT 1 hour	0.41 (95% CI: 0.21, 0.83)	.034				
	OGTT 2 hour	0.37 (95% CI: 0.17, 0.82)					
	OGTT 3 hour	0.22 (95% CI: 0.06, 0.88)					
	2+ vs 1 abnormal			2.89 (95% CI: 2.05, 4.08)	<.001		
	No of abnormal					2.01 (95% CI: 1.65, 2.45)	<.001
Race/ethnicity	Asian	3:70 (95% CI: 0.86, 3.4)	.224	1.56 (95% CI: 0.97, 2.50)	.359	1.48 (95% CI: 0.91, 2.40)	.457
	Black	0.78 (95% CI: 0.26, 2.35)		1.38 (95% CI: 0.76, 2.51)		1.27 (95% CI: 0.69, l 2.34)	
	Hispanic	1.06 (95% CI: 0.50, 2.22)		1.34 (95% CI: 0.83, 2.16)		1.32 (95% CI: 0.82, 2.14)	
	Other/multiracial	2.37 (95% CI: 0.94, 5.95)		1.47 (95% CI: 0.79, 2.75)		1.50 (95% CI: 0.81, 2.80)	
BMI		1.09 (95% CI: 1.05, 1.14)	<.0001	1.08 (95% CI: 1.05, 1.11)	<.0001	1.08 (95% CI: 1.05, 1.11)	<.0001
Gestational age (completed weeks) at time of OGTT, median (IQR)		0.96 (95% CI: 0.86, 1.07)	.4781	0.90 (95% CI: 0.83, 0.97)	.0040	0.90 (95% CI: 0.84, 0.97)	.0048
Maternal age at delivery		1.04 (95% CI: 0.98, 1.11)	.1824	1.03 (95% CI: 0.99, 1.07)	.1019	1.03 (95% CI: 0.99, 1.07)	.0990
Parity		1.01 (95% CI: 0.78, 1.32)	.9162	0.97 (95% CI: 0.83, 1.14)	.7280	0.99 (95% CI: 0.84, 1.17)	.9232
Prior GDM		2.64 (95% CI: 1.15, 6.07)	.0219	1.10 (95% CI: 0.67, 1.82)	.7079	1.04 (95% CI: 0.62, 1.74)	.8949
Prior macrosomia		0.69 (95% CI: 0.22, 2.17)	.5269	1.41 (95% CI: 0.72, 2.77)	.3135	1.29 (95% CI: 0.65, 2.57)	.4632
AUC		0.71		0.72		0.72	

Abbreviations: aOR, adjusted odds ratio; AUC, area under the curve; BMI, body mass index; GA, gestational age; GDM, gestational diabetes mellitus; IQR, interquartile range; OGTT, oral glucose tolerance test.

Model 1 included categorical OGTT values applied to the 1-abnormal group.

Model 2 included binary abnormal OGTT values (≥2 abnormal vs 1 abnormal).

Model 3 included linear number of abnormal OGTT values.

*Intercept values represent odds when all variables are at baseline values (OGTT = fasting or 1 abnormal depending on model; race/ethnicity = White; BMI = 28.7; GA at OGTT = 28.3; maternal age at delivery = 33.2; nulliparous; no history of GDM or macrosomia).

**Table 4:** Fetal and neonatal outcomes by number of elevation(s) on glucose tolerance test and need for pharmacotherapy

Outcome	Treatment group	1 Abnormal value	2+ Abnormal values	*P* value	RR
Polyhydramnios	Overall	7.0% [N = 445]	2.6% [N = 341]	.009	2.64 [1.27, 5.47]
	Dietary management only	7.2% [N = 361]	3.0% [N = 198]	.051	2.38 [1.00, 5.68]
	Pharmacotherapy	6.0% [N = 84]	2.1% [N = 143]	.146	2.84 [0.70, 11.57]
Large for gestational age	Overall	13.1% [N = 442]	13.6% [N = 337]	.830	0.96 [0.67, 1.38]
	Dietary management only	11.7% [N = 358]	11.3% [N = 194]	.891	1.03 [0.64, 1.68]
	Pharmacotherapy	19.0% [N = 84]	16.8% [N = 143]	.665	1.13 [0.64, 2.01]
Hypertensive disorders of pregnancy	Overall	15.1% [N = 443]	12.7% [N = 339]	.333	1.19 [0.84, 1.70]
	Dietary management only	15.0% [N = 359]	13.3% [N = 195]	.586	1.13 [0.73, 1.74]
	Pharmacotherapy	15.5% [N = 84]	11.8% [N = 144]	.429	1.31 [0.67, 2.56]
Neonatal complications	Overall	23.5% [N = 442]	16.0% [N = 338]	.011	1.47 [1.09, 1.98]
	Dietary management only	22.1% [N = 358]	13.8% [N = 195]	.023	1.59 [1.07, 2.38]
	Pharmacotherapy	29.8% [N = 84]	18.9% [N = 143]	.059	1.58 [0.98, 2.53]

Abbreviation: RR, relative risk.

Neonatal complications include shoulder dystocia, neonatal intensive care admission, neonatal hypoglycemia, and hyperbilirubinemia

**Figure 1:**
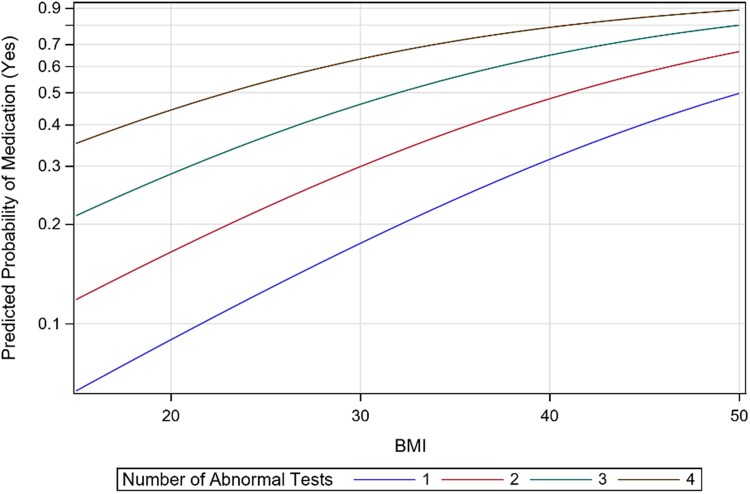
Risk of requiring medication based on maternal BMI and number of abnormal OGTT values.

We further refined the odds of requiring pharmacotherapy by incorporating the number of abnormal OGTT results. The odds ratio associated with each additional abnormal result was equivalent to the odds ratio of a change in BMI of 9.05 (CI 6.04, 14.87) when the covariables were held fixed (Fig. 1). The risk formula itself evaluates all 4 timepoints of the OGTT, so values below the cutoff for a given time point will lower the predicted risk.

We developed a prediction model that combines maternal BMI and the degree of normality of the 4 OGTT results using linear discriminant analysis. The reference data showed a significant difference between the medication and non-medication groups for degree of normality (value minus cutoff) of elevation with fasting (*P* < .001), at 1 hour (*P* = .005), at 2 hours (*P* < .001), and at 3 hours (*P* < .001) (16). Allowing for an overall 10% positive rate (an increase of 13.2% in number of high-risk patients compared to 2-elevated OGTT) resulted in 15.4% increase in the number of true gestational diabetes cases identified. We evaluated the accuracy of the model using a calibration plot that compares the average observed incidence of need for pharmacotherapy to the average predicted risk for need for pharmacotherapy (17). The model fit well except at extremely high-risk values.

## Discussion

### Key results

This study demonstrates that almost 20% of patients with 1 OGTT elevation required pharmacotherapy for glycemic control. Among patients with 1 elevation on OGTT, patients with fasting elevations are more likely to need pharmacotherapy compared to those with elevations following administration of a 100-gram glucose load. Elevations occurring at longer durations from administration of OGTT were not associated with increased need for pharmacotherapy. Body mass index and extent of glucose elevation above the threshold are additional important risks for need for pharmacotherapy, with patients with BMI over 40 and increasing glucose values on OGTT being likely to require pharmacotherapy regardless of number of abnormal glucose values.

We used maternal BMI, number of glucose elevations, and extent of glucose elevations on OGTT to create a predictive model of a priori risk for pharmacotherapy in patients with 1 or more elevation on OGTT. This model is more accurate at predicting need for pharmacotherapy than the timing of elevation on OGTT alone. Predictive models may have utility in assessing need for pharmacotherapy for patients with an abnormal OGTT, particularly those with 1 elevation on OGTT. Additional research with a larger sample size is indicated to create a regression analysis calculator that can assess a patient's likelihood for need for pharmacotherapy for glycemic control similar to the vaginal birth after cesarean calculator (18) or the Extremely Preterm Birth Outcomes tool (19).

### Interpretation

Predictive models exist to evaluate the risk of developing gestational diabetes among the general obstetric population (20). These require machine learning capabilities and large datasets, which may not be feasible or accessible for all prenatal patients. Other studies utilize predictive models to identify the need for pharmacotherapy among patients with 2 or more OGTT elevations (19, 21), with baseline blood glucose and elevated maternal BMI similarly increasing the probability of requiring pharmacotherapy (21). One small retrospective study that assessed patients with “mild” gestational diabetes, defined as fasting plasma glucose <95 mg/dL, conversely did not find antenatal factors that reliably predicted the need for pharmacotherapy (22). However, to our knowledge, there are no published predictive models that assess need for pharmacotherapy among patients with glucose intolerance. This study is unique in that it uses readily available clinical demographics to identify patients at increased risk for pharmacotherapy who would otherwise not be monitored or treated in usual obstetrical practice. This study is also unique in that it assesses not only the number of glucose elevations, but the extent of elevation(s) above a set threshold.

The association between fasting hyperglycemia on OGTT and need for pharmacotherapy is consistent with prior literature of predictive fasting elevations (23) and suggests that these patients may be at higher risk for development of medication-requiring gestational diabetes. Fasting hyperglycemia in patients with gestational diabetes is an indirect marker of increased insulin resistance from the influence of placentally derived circulating hormones, particularly in the second and third trimesters, and is a result of physiologic adaptation to pregnancy to support the advancing gestation (24). Another study similarly demonstrates that patients with an abnormally elevated 1-hour value on OGTT have similar outcomes to patients with classically defined gestational diabetes, whereas patients with a 2-hour or 3-hour elevation on OGTT behave more similarly to patients with normal glucose tolerance (25). The increasing need for pharmacotherapy with increasing number of elevations on OGTT has been well-characterized (26) and may be considered a predictive factor at time of OGTT analysis.

Our study demonstrated increased risk of composite neonatal complications among patients with 1 elevation on OGTT for those managed with diet alone but not for those requiring pharmacotherapy, and similarly increased risk of polyhydramnios overall in the group with one elevation on OGTT that was not significant in the group that required pharmacotherapy. These data are suggestive of risk mitigation with introduction of pharmacotherapy for glycemic control.

In the existing literature, pharmacotherapy was associated with decreased risk of metabolic complications and large neonates among patients with one abnormal value on OGTT (27). We hypothesize that patients with 1 elevation on OGTT were treated with pharmacotherapy less aggressively than those with 2 or more elevations, leading to increased risk of obstetric complications such as polyhydramnios and neonatal complications. While there were differing patterns of pharmacotherapy between patients with 1 OGTT elevation and 2 or more OGTT elevations, interestingly, more patients with 1 elevation received insulin pharmacotherapy; therefore, differing prescribing patterns do not describe these differences alone. Glycemic monitoring and treatment of hyperglycemia has been associated with neonatal benefit; a prospective controlled trial that compared treatment for 1 elevation on OGTT to no treatment found that the overall incidence of neonatal metabolic complications and large infants was significantly lower in the treated group (27); more research is indicated to evaluate whether the type of antihyperglycemic medication affects the likelihood of polyhydramnios, similar to the association of glyburide with fetal macrosomia compared to insulin or metformin (28). Our findings, in the context of the existing literature, suggest that there may be antenatal and neonatal clinical benefits of glycemic monitoring and treatment when indicated in patients with an abnormal OGTT without overt gestational diabetes.

Our findings are clinically relevant, given that patients who require pharmacotherapy are recommended antenatal testing at an earlier gestational age and are recommended delivery at an earlier gestational age compared to patients managed on diet and lifestyle modification alone (1). However, in most clinical centers, patients with only 1 elevation on OGTT are typically not followed with antenatal glucose monitoring. Our findings suggest that a subset of patients with only 1 elevation on OGTT (those with fasting or 1-hour elevations and those with increased BMI) are more likely to require pharmacotherapy, which is a surrogate for hyperglycemia in pregnancy. Our definition for gestational diabetes (1 abnormal 100 g antenatal OGTT value) that other authors have previously referred to as “gestational glucose intolerance” reflects the view that the current “2-abnormal-OGTT-values” criteria of O'Sullivan are insufficiently sensitive.

Future directions include evaluation of cost analysis of management and treatment of patients with 1 elevation on OGTT and expanded use of prediction models to predict with improved accuracy the need for pharmacotherapy among patients with at least 1 elevation on OGTT to stratify antepartum risks and guide clinical management.

### Strengths and limitations

The main strength of this study is the access to data of outcomes for patients followed with abnormal glucose levels on OGTT who do not otherwise meet criteria for gestational diabetes. Our predictive model's strength is its accessibility without the need for expansive datasets and/or machine learning capabilities. A limitation to the development of this model is selection bias due to the exclusion of patients with no elevations on 3-hour OGTT given lack of available glucose monitoring and data, which may decrease the differences between the medication and non-medication groups. In addition, further studies focused on different risk prediction algorithms (for example, logistic regression or machine learning) are needed to determine the best algorithm to calculate risk.

A key limitation of this study is the choice of this primary outcome as the threshold at which to initiate medication that is not standardized nationally or institutionally. As is widely the case, there is no concrete definition of “poor glycemic control” warranting initiation of pharmacotherapy. We acknowledge that other protocols may have more strict definitions of suboptimal or poor glycemic control, thereby limiting the generalizability of this study which relies on an institution-specific protocol. Additional limitations include the retrospective design and small sample size, particularly for patients with 1 fasting elevation on OGTT and patients with a 3-hour elevation. Use of search terms to determine eligible patients in ASOBGyn relies on codes input by providers, including physicians and sonographers, to identify eligible patients. Additionally, our study included patients who received oral pharmacologic therapy, where it is known that insulin is the gold standard pharmacologic therapy for treatment of gestational diabetes.

## Conclusions

This retrospective cohort study examines the need for pharmacotherapy for patients with glucose intolerance defined as having at least 1 elevation on OGTT. Although patients with 2 or more glucose elevations on OGTT were more likely to require pharmacotherapy for glycemic control, patients with a single elevation were more likely to develop polyhydramnios and neonatal complications; however, this latter outcome was not significant among patients treated with pharmacotherapy. Patients with only 1 OGTT elevation with high BMI, elevated fasting value on OGTT, or 1 abnormal OGTT value that greatly exceeds the testing threshold may benefit from finger stick monitoring to screen for hyperglycemia in pregnancy.

## Data Availability

Original data generated and analyzed during this study are included in this published article or in the data repositories listed in “References.”

## Abbreviations

BMI: body mass index
GCT: glucose challenge test
NICU: neonatal intensive care unit
OGTT: oral glucose tolerance test
